# Clinician Decision Making in Managing Primary Aldosteronism: A Qualitative Study

**DOI:** 10.1016/j.xkme.2026.101429

**Published:** 2026-06-12

**Authors:** Sandra Hakim, Lok Him Jason Yeung, Grant Russell, Lisa Dubrofsky, Gregory Kline, Jun Yang, Katrina M. Long

**Affiliations:** 1 School of Primary and Allied Health Care, Monash University, Frankston, VIC, Australia; 2 National Centre for Healthy Ageing, Frankston Hospital, Frankston, VIC, Australia; 3 Peninsula Health, Frankston Hospital, Frankston, VIC, Australia; 4 Department of Occupational Therapy, School of Primary and Allied Health Care, Monash University, Frankston, VIC, Australia; 5 Department of General Practice, Monash University, Melbourne, VIC, Australia; 6 Department of Medicine, Women’s College Hospital, Toronto, ON, Canada; 7 Department of Medicine, Cumming School of Medicine, University of Calgary, Calgary, AB, Canada; 8 Centre for Endocrinology and Reproductive Health, Hudson Institute of Medical Research, Clayton, VIC, Australia; 9 Department of Medicine, Monash University, Clayton, VIC, Australia

**Keywords:** Clinical decision making, clinical management, primary aldosteronism, qualitative research, secondary hypertension

## Abstract

**Rationale & Objectives:**

Primary aldosteronism (PA) is underdiagnosed in people living with hypertension, despite its considerable prevalence, association with poorer cardiovascular and kidney outcomes, and availability of effective treatment. The factors underlying such underdiagnosis and undertreatment remain uncertain. This study aimed to identify key factors influencing screening, diagnosis and treatment of PA from the clinician’s perspective.

**Study Design:**

Exploratory qualitative study.

**Setting and Participants:**

Thirty-eight clinicians were recruited from across Australia, including cardiologists (n = 8), endocrinologists (n = 10), general practitioners (n = 10) and nephrologists (n = 10). Purposive sampling achieved diversity in practice location, setting, and clinical experience. In-depth semistructured interviews were conducted between May and November 2024.

**Analytical Approach:**

Interview transcripts were analyzed using both deductive content analysis aligned with the Consolidated Framework for Implementation Research and inductive reflexive thematic analysis.

**Results:**

Four themes were developed to explain factors affecting clinician screening, diagnosis, and treatment decisions: (1) Clinician experience, knowledge, and perceptions; (2) PA screening and diagnosis complexity and burden; (3) accessibility of services and information; and (4) health system, government, and organizational factors. Common barriers across clinician groups included that PA is often considered in a narrow patient cohort, with older and comorbid populations not considered candidates for screening; that the perceived complexity and burden associated with PA diagnosis inhibits initiation of the diagnostic process; that limited access to services is a barrier to screening or workup of PA; and that lack of streamlined and local diagnostic services contributes to fragmented care and diagnostic delay. Key facilitators included access to specialized diagnostic units and cross-disciplinary and collegial decision making in patient management.

**Limitations:**

Generalizability may be limited due to Australian context.

**Conclusions:**

These findings highlight key factors that influence clinicians’ decision making in PA detection and management, providing important guidance to the design of future clinician-targeted interventions to improve PA diagnosis and treatment.

## Plain-Language Summary

Primary aldosteronism (PA) is the most common hormonal cause of hypertension; however, it is vastly underdiagnosed and often suboptimally managed. The basis for this is poorly understood. To explore this, nephrologists, endocrinologists, cardiologists, and general practitioners were interviewed to understand their decision making in PA management. Major barriers included: often only narrow profiles of patients were considered candidates for PA screening; the diagnostic process was perceived as burdensome and therefore often not initiated; and that limited diagnostic service access dictated clinical management and contributed to diagnostic delay. Conversely, specialized diagnostic units and professional networks promoted optimal patient management. These findings highlight key factors that can be leveraged in the design of future clinician-targeted interventions to improve PA care.

## Graphical abstract

**Figure:**
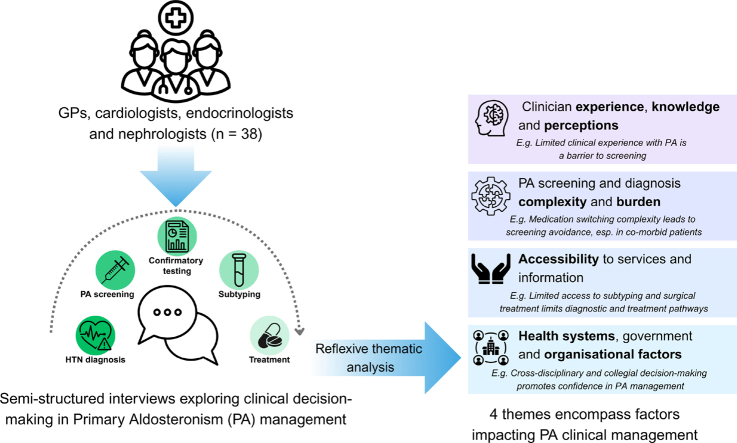

In Australia, an estimated 34% of adults have hypertension^1^ contributing to 5.1% of the total burden of disease.^2^ Of patients with hypertension, an estimated 3.2%-14% have primary aldosteronism (PA),^3^^,^^4^ a potentially curable condition of excess aldosterone production associated with 2- to 4-fold higher risk of coronary artery disease, atrial fibrillation, and stroke.^5^, ^6^, ^7^, ^8^

PA can be detected using the aldosterone-renin ratio (ARR), a simple blood test; however, screening rates remain very low. European studies have shown only 3%-7% of hypertensive patients in primary care are ever screened for PA,^9^^,^^10^ while Canadian and Australian studies revealed screening rates of <1%.^11^^,^^12^ Underdiagnosis and/or diagnostic delay contributes to potentially preventable kidney damage,^13^ cardiovascular complications, and end-organ damage.^14^^,^^15^

Although ARR testing is the first step, the diagnostic pathway for PA is complex and often requires specialist review, confirmatory testing, and assessment for surgical treatment.^16^ Despite this complexity, consumer survey data suggest that people are highly motivated to pursue testing to identify the root cause of their hypertension^17^ but experience diagnostic delay, which is associated with poor outcomes.^15^

Few studies have examined clinician perspectives on PA screening, diagnosis, and treatment, particularly beyond initial screening or the primary care context. To date, only 1 in-depth, qualitative study has reported barriers to PA screening for Australian general practitioners (GPs).^18^ Given that hypertension is managed by various specialties, there is a need to understand the factors affecting PA screening and diagnosis, as well as treatment decisions, across the main disciplines that manage hypertension. This study therefore explored clinician decision making in PA screening, diagnosis, and treatment, across specialties involved in hypertension care (GPs, nephrologists, endocrinologists, and cardiologists). Specifically, this study aimed to identify factors influencing decisions to screen, investigate, and treat PA.

## Methods

### Study Design

A qualitative, exploratory study was chosen to allow in-depth investigation of clinicians’ experiences and perspectives on barriers and facilitators to the screening, diagnosis, and treatment of PA. A semistructured interview guide was developed using the updated Consolidated Framework for Implementation Research Interview Guide Tool,^19^ a commonly used implementation science framework^20^^,^^21^ (Item S1). This study is reported in accordance with the Consolidated Criteria for Reporting Qualitative Research^22^ (for full methods, see Item S2).

### Recruitment and Sampling

Participants were recruited using purposive and snowball sampling and included endocrinologists, nephrologists, cardiologists, and GPs practicing in Australia. Clinicians involved in PA research as a chief investigator, associate investigator, sponsor, or participant within the past 5 years were excluded. Purposive sampling ensured diverse representation across key characteristics (specialty, location, setting, clinical experience). Snowball sampling was achieved via interview participants and members of the Primary Aldosteronism Foundation and its associated Facebook group. Sampling continued until theoretical saturation was reached. Advertising occurred via professional networks and health services. Study advertisements were also directed at people living with PA, inviting them to nominate a clinician for the study via a nomination survey. Potential participants were invited to complete a screening survey to confirm eligibility. Both surveys were hosted on Qualtrics XM (Qualtrics).

### Data Collection and Analysis

Semistructured interviews were conducted via Zoom (Zoom Video Communications, Inc) from May to November 2024. Transcripts were generated using Panopto (version 15.9.0.00022) and analyzed using NVivo (version 14; Lumivero). Two complementary qualitative analysis approaches were employed simultaneously: deductive content analysis, to code interview content against Consolidated Framework for Implementation Research domains^19^ and Endocrine Society guidelines^16^; and reflective thematic analysis,^23^ to inductively explore clinicians’ experiences and approaches to diagnosing and managing PA.

### Ethics Approval and Consent

This study was approved by the Monash University Human Ethics Committee (Project ID: 41728). All participants were provided with a full explanatory statement. Consent was implied via completion of the screening survey and verbally reaffirmed at the beginning of interviews.

## Results

### Participant Demographics

**Figure 1:**
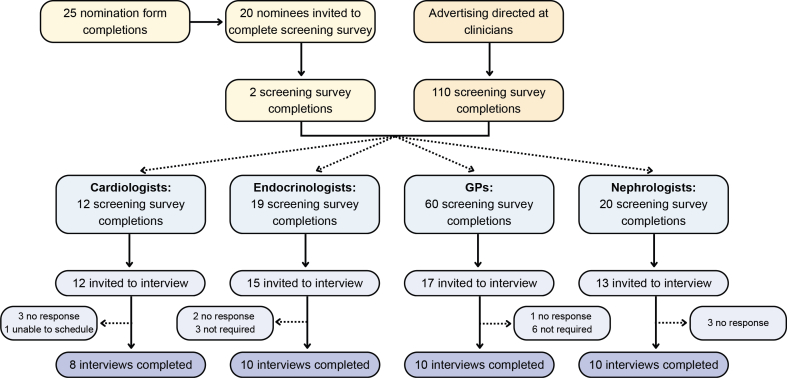
Study recruitment. Patient- and clinician-directed advertising led potential participants to complete the screening survey. Individuals invited to interviews were no longer required when saturation for clinician groups was achieved. GP, general practitioner.

A total of 38 clinicians completed interviews, including 8 cardiologists, 10 endocrinologists, 10 GPs, and 10 nephrologists (Fig 1). Interviews ranged from 23-69 minutes (average 46 min 21 s). Seventeen participants reviewed a preliminary report of study results with no major changes to data interpretation. Fewer cardiologists were recruited due to lower expressed interest, whereas interest from other specialties exceeded study capacity, enabling greater participant diversity. Participant demographics stratified by specialty are shown in Table S1. Across all specialties, a broad range of clinical experience and practice settings were represented. Participants were located across all Australian states and territories (Victoria and New South Wales were most highly represented) and across metropolitan, regional, and remote locations.

### Clinical Roles and Pathways in PA Management

**Figure 2:**
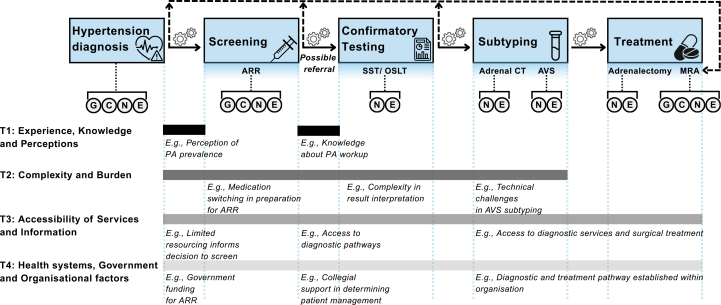
Clinical management of PA by specialty and inductive themes. The clinical pathway of PA management. Key decisions are indicated by the cog graphic. Dashed arrows represent alternative pathways to MRA treatment. Steps performed by different specialties are indicated within circles (G: general practitioners; C: cardiologists; N: nephrologists; E: endocrinologists). Contributions of identified themes at various stages of PA management are represented by shaded bars. Abbreviations: ARR, aldosterone-renin ratio; AVS, adrenal vein sampling; CT, computed tomography; MRA, mineralocorticoid receptor antagonist; PA, primary aldosteronism; OSLT, oral salt loading test; SST, saline suppression test.

Reported roles of different specialties in PA management were mapped to Endocrine Society guidelines.^16^ GPs reported predominantly ordering ARRs and referring patients with abnormal results to endocrinologists, while managing medications in confirmed cases. Cardiologists predominantly ordered ARRs, initiated mineralocorticoid receptor antagonists (MRAs), and/or referred to endocrinologists for further evaluation. Nephrologists and endocrinologists undertook similar roles in managing PA, from screening to treatment. Those with access to specialized endocrine hypertension services referred patients to those services for management rather than actively coordinating care themselves (Fig 2).

### Factors Influencing Clinician Decision Making in PA Screening and Management

Findings were similar across specialties; therefore, results are reported collectively. Deductive analysis (Table S2) revealed consistent code representation across specialties. Codes were organized into the inductive framework to establish a conceptual model of factors influencing decision making in PA management (Fig 2). The inductive coding matrix (Table S3) revealed similar patterns across specialties.

Four overarching themes emerged that influenced clinician decision making: (1) clinician experience, knowledge, and perceptions; (2) burden and complexity; (3) accessibility of services and information; and (4) government and organizational factors (Box 1). The 4 themes encompass various aspects of the screening and management pathway for PA (Fig 2). Themes 3 and 4 influenced all stages of the PA management pathway, whereas theme 2 primarily affected screening and confirmatory testing, and theme 1 most strongly influenced decisions to screen or pursue diagnostic workup.

Box 1 **Inductive Themes**

**Table:** 

Theme	Subtheme	Category
T1 – Clinician experience, knowledge, and perceptions guide clinical decision making in PA screening and management	Clinical exposure to PA	Clinical experience informs practice and further education
		Limited clinical exposure to PA or full workup to adrenalectomy
		Positive clinical experience with procedure or treatment
	Educational sources and guidelines	Reference to clinical guidelines or online resources
		Education source
		Influential figures
		Lack of information for clinicians
	Perceptions about PA, patients and other clinicians	Clinician perceptions about PA
		Perception of patient health literacy and compliance
		Perception of other clinicians
	Clinical criteria and judgment for patient management	Patient characteristics and clinical information that inhibits secondary HTN or PA screening or PA workup
		Patient characteristics and clinical information that promote secondary HTN or PA screening or PA workup
		Routine practice for clinicians in secondary HTN screening
T2 – PA screening and diagnosis complexity and burden influences clinician decision making	Clinical guideline complexity, test reliability, and technical challenges	Clinical guideline complexity and challenges
		Test reliability
		Technical challenges
		Test or procedure is acceptable or uncomplicated
	Patient complexity and medication switching	Patient comorbid conditions and polypharmacy
		Variability in patient clinical manifestation and circumstances
		Medication switching burden
		Medication switching complexity
	Burden vs advantage in pursuing PA diagnosis	Advantage of PA diagnosis (general)
		Advantage of PA diagnosis for younger patients
		Conditions must be met for PA screening or workup to proceed
		Perception of limited benefit in PA screening or workup
		Adverse events promote PA investigation and treatment
T3 – Accessibility of services and information influence clinical management	Access to medical facilities and services	Ease of access to services
		Limited or lack of access to services
		Limited access to primary care
		Limited resourcing affects screening and treatment decisions (triage) and outcomes
		Referrals and follow-up
		Time allocation
		Accessibility of guidelines and resources
	Challenges navigating patient preferences, safety, and cost	Patient preferences and needs
		Safety and tolerability of procedures or treatments
		Cost to patient
T4 – Health systems, government, and organizational factors underpin screening, diagnosis, and treatment decisions	Health systems, personnel, and patient management	Specialized services
		Implementation facilitators
		Disjointed care and delayed diagnosis
		Patient follow-up in hospital setting
		Challenges in patient management
		Procedural facilitators in PA management
	Government funding for tests, services, and treatments	Services that facilitate PA diagnosis and management
		Medicare and Pharmaceutical Benefits Scheme-related barriers to PA diagnosis and management
		Economic factors
	Communication and professional networks	Clinician–clinician and patient correspondence
		Organizational meetings
		Referrals
		Limited communication between clinicians
		Isolation in clinical practice
	Professional role and culture	Workplace exposure and clinical role
		Perceptions of the role of other specialties in PA management
		Assumed roles in PA diagnosis and management

Abbreviations: HTN, hypertension; PA, primary aldosteronism.

### Theme 1: Clinician Experience, Knowledge, and Perceptions Guide Clinical Decision Making in PA Screening and Management

Knowledge and perceptions about PA and different patient populations contributed to clinical decision making in PA screening and management.

#### Clinical Exposure to PA

Clinical exposure to PA cases strongly shaped clinicians’ perceived value of screening and confidence in management. Many described experience-based knowledge from current or past roles that influenced their willingness to screen for PA (Table 1, Q1.1). Previous clinical exposure also promoted self-directed education about PA, whereas lack of recent exposure contributed to outdated knowledge about PA (Table S4, Q1.1). Limited clinical encounters with PA, including limited exposure to the full screening-to-treatment pathway, was reported across specialties (Table S4, Q1.2). This led some to question the utility of screening, even when aware of PA prevalence (Table 1, Q1.2).

**Table 1:** Theme 1 Representative Quotes

Theme 1: Clinician Experience, Knowledge, and Perceptions Guide Clinical Decision Making in Primary Aldosteronism (PA) Screening and Management		
Subtheme	Label	Quote
Clinical exposure to PA	Q1.1	“I think seeing the few cases where [PA] was diagnosed, and then once they got the right treatment…things got better. And that’s quite reassuring…in the past you think about younger people…but seeing these new diagnoses in older individuals…made me realize that the pick-up rate is high enough that you think it’s worthwhile doing. There are other tests that we do, where the pick-up rate is much lower…so it would make sense to do [it].”- N08
	Q1.2	“You know, I’ve seen figures quoted as quite a high incidence of PA. I think even 8% of hypertensive [patients]…which makes me wonder whether I should be doing an ARR as a first line investigation as opposed to a second line investigation. But I don’t think I’ve seen the numbers in my own practice that make me suspect that it is actually as high as quoted. So, I’m still not completely convinced that that it should be a first line investigation."- G11
Educational sources and guidelines	Q1.3	"[Victorian health care provider]…absolutely excels in this because of the endocrine unit with an interest in [PA] headed by [influential figure 1] and [influential figure 2]…[I] have benefited from the excellent guidance of those individuals…It’s been really well described by [influential figure 1] in particular, at various meetings…making this is a user-friendly pathway.”- E01
	Q1.4	“I really don’t think that we get good teaching and education about [PA], even on a specialist level, and that’s part of the problem.”- C02
Perceptions about PA, patients, and other clinicians	Q1.5	“I’d say it’s much more common to have hypertension from metabolic disease where we live, from obesity, poor diet, no exercise, and there’s lots of sleep apnea here…a lot of the other causes are much more pressing and prevalent, which shouldn't take away from the fact that there’s a probably a lot more aldosteronism than we know of, because we’re not testing for various reasons.”- E09
	Q1.6	“Because [medication switching] is sufficiently complicated, I think you’d be looking for a patient who is young and appears to have a good grasp of their health care as well…it’s quite hard unless you either have a patient who knows what they’re doing or a patient who has a carer who is able to help…”- C01
Clinical criteria and judgment for patient management	Q1.7	“I think children would be one cohort I might not investigate, or…much older adults…[over] 70s…I might not think of primary aldosteronism as the first thing. I might think about cardiovascular stuff like atherosclerosis…I find it much trickier when someone’s already on medications for a long time and their hypertension is well controlled…to try to reinvestigate things…I might have to change my thinking around this.”- G05

#### Educational Sources and Guidelines

Most participants reported accessing clinical guidelines or online resources to guide PA screening and management. Many also reported that meetings, conferences, clinical training, engagement in research, and influential figures or institutions specializing in PA informed their management of PA (Table 1, Q1.3). However, some reported having inadequate information, particularly to guide confirmatory testing (Table S4, Q1.3). Limited coverage in medical training was also identified as a barrier (Table 1, Q1.4).

#### Perceptions About PA, Patients, and Other Clinicians

Perceptions of PA influenced decisions to pursue diagnosis. Although some were motivated by awareness of PA prevalence and risks (Table S4, Q1.4), others perceived PA as rare or not a major contributor to disease in their specific patient demographic, limiting the impetus to screen (Table 1, Q1.5).

Perceptions about patient health literacy and compliance also influenced screening (Table S4, Q1.5). Specifically, older, culturally and linguistically diverse, and comorbid patients were perceived as having limited capability to manage medication switching or adherence to screening protocols (Table 1, Q1.6).

Participants also reported perceptions of other clinicians’ management of PA. Most described barriers for GPs to perform screening, including limited consult time, competing priorities, and lack of PA-specific education (Table S4, Q1.6). Some participants held the view that recent graduates were more likely to screen for PA (Table S4, Q1.7).

#### Clinical Criteria and Judgment for Patient Management

Decisions to screen for secondary causes of hypertension, including PA, were often guided by clinical acumen, with most clinicians restricting PA testing to selected patients (eg, younger, taking multiple antihypertensive medications, evidence of electrolyte abnormalities, and end-organ damage) (Table S4, Q1.8). Many participants did not consider screening older hypertensive patients or those with pre-existing alternative diagnoses (including essential hypertension and complex comorbid conditions), or evidence of lifestyle factors that could contribute to hypertension (Table 1, Q1.7).

Family history of hypertension was also considered by many participants when deciding to perform screening, although whether this promoted or inhibited screening was variable. Only a minority routinely screened all newly diagnosed hypertensive patients for secondary hypertension, including PA.

### Theme 2: PA Diagnosis and Management Complexity and Burden Influences Clinician Decision Making

Many participants considered PA as burdensome to diagnose due to complex patient presentations and diagnostic processes. Many participants therefore made judgments on the value of pursuing PA diagnosis to inform their decision making.

#### Clinical Guideline Complexity, Test Reliability, and Technical Challenges

Although many participants reported accessing clinical guidelines to inform decision making for secondary hypertension screening and management, there were opposing views relating to their acceptability and utility. Some participants reported that current guidelines were complicated, decentralized, or impractical (Table S5, Q2.1), whereas a similar proportion considered guidelines acceptable or uncomplicated (Table S5, Q2.2).

Many participants reported concerns regarding the reliability of PA screening and confirmatory testing, due to factors including patient clinical variability, heterogeneous test thresholds, and interfering medications that may lead to false results (Table 2, Q2.1). Technical challenges in PA diagnosis were also reported, including delays in receiving test results, multiple tests and procedures required to achieve a diagnosis, operator errors during adrenal vein sampling (AVS; such as failed adrenal vein cannulation), and MRA titration with management of hyperkalemia (Table S5, Q2.3). However, some participants expressed confidence in PA diagnostic or treatment interventions, particularly measuring the ARR (considered uncomplicated and compatible with other blood tests) (Table S5, Q2.4), point of care cortisol testing during AVS (validating cannulation success), or pathology processing via reputable organizations, all leading to confidence in screening/diagnosis. Adrenalectomy was also considered uncomplicated and highly effective.

**Table 2:** Theme 2 Representative Quotes

Theme 2: PA Diagnosis and Management Complexity and Burden Influence Clinician Decision Making		
Subtheme	Label	Quote
Clinical guideline complexity, test reliability, and technical challenges	Q2.1	“There's patients in hospital…we can measure [ARR], but half the time they have been walking around the minute prior to getting their blood test, and it’s a little bit diabolical as to what any of the results even mean sometimes, and they might be on some of the medications that affect the interpretation.”- E09
Patient complexity and medication switching	Q2.2	“Sometimes it’s quite long [before initiating a secondary screen] because I'm seeing [the patient] about a primary issue, it might come up as the secondary issue. This person is on potassium and three antihypertensives, this probably needs to be looked at, but…let’s deal with what they’ve come to me about first…If it doesn’t seem like the current active, uncontrolled issue, then it could be put on the back burner over a long period of time.”- E02
Burden versus advantage in pursuing PA diagnosis	Q2.3	“It’s tricky when they’ve come in on medication, so then I decide, am I going to presume that they’ve got [PA] and give them an MRA, or am I going to wash them out and swap to something temporary for six weeks? I do occasionally for those younger ones, because I do want to know…it’s nice to give them a reason why.”- C08

Abbreviations: ARR, aldosterone-renin ratio; MRA, mineralocorticoid receptor antagonist; PA, primary aldosteronism.

#### Patient Complexity and Medication Switching

Patient comorbid conditions, polypharmacy, variable clinical manifestation, and variability in circumstances of presentation often delayed or obscured PA investigation (Table 2, Q2.2). Medication switching was considered challenging in comorbid patients on multiple medications due to safety concerns and adherence to alternative medications. The necessity to titrate alternative mediations and monitor blood pressure during this period was also viewed as burdensome (Table S5, Q2.5). These challenges were associated with a tendency to avoid screening in this patient population by some participants.

#### Burden Versus Advantage in Pursuing PA Diagnosis

Although some participants articulated the value of PA screening and diagnosis, many reported reluctance to screen or pursue diagnosis due to a perception that burden outweighs benefit, particularly in older populations, with management unlikely to change and uncertain benefit of MRAs compared with existing medications (Table 2, Q2.3). Some preferred empirical MRA treatment without screening, especially when a surgical cure was deemed unlikely (Table S5, Q2.6).

A small number of participants indicated that serious clinical events, such as hypertensive crisis, end-organ damage, and intolerable side effects from MRA treatment, promoted PA investigation that would not otherwise have occurred (Table S5, Q2.7).

### Theme 3: Accessibility of Services and Information Influence PA Diagnosis and Management

Access to appropriate services and resources was identified as a major determinant of decisions regarding PA screening and management.

#### Access to Medical Facilities and Services

Although some participants were satisfied with access to medical facilities/specialist services for PA diagnosis and treatment, many reported access challenges. Lack of availability of AVS and saline suppression testing, including due to lack of staff expertise, was most commonly reported in regional/remote areas (Table S6, Q3.1) but also in metropolitan areas. These resource constraints often necessitated triaging patients for further investigation (Table 3, Q3.1).

**Table 3:** Theme 3 Representative Quotes

Theme 3: Accessibility of Services and Information Influence PA Diagnosis and Management		
Subtheme	Label	Quote
Access to medical facilities and services	Q3.1	“I know the Melbourne experience is different, but we’re resource poor [in NSW metropolitan city]. And so, trying to pick and choose which patients need investigations and invasive testing versus don't need.”- E03
	Q3.2	“All the inpatient stuff runs quite well. The outpatient service is just completely overwhelmed…I think the obvious answer is to say we need more clinic space…more staff, but the primary care setting…particularly [in] regional areas around the country…seems to be completely collapsing…People can't see a GP, or they won’t…because they can’t afford it, and then they end up…in our service, at the last minute…The volume of referrals has just exploded for everything in the last couple of years…We’ve got about 2,500 people on our outpatient waiting list.”- C03
	Q3.3	“I think particularly being in a regional area, it’s really important that I'm proactive about testing and not trying to find the optimal circumstances to do it, because people just leave hospital and get lost to follow up all the time…They find a lot of difficulty actually having a consistent GP service, particularly in the peripheral centers. So, I order [ARR] off the bat if they’re hypertensive and I’ve got a suspicion based on their pre-test probability…I’ll also communicate with the GPs where possible, to withdraw any culprit or interfering medications and retest in six weeks…But a lot of the time, that’s pretty hard. And that that’s…a reflection of the sparsity of health care that we have out here, more than anything."- E06
Challenges navigating patient preferences, safety, and cost	Q3.4	“I’m aware that there’s a group of drugs that [patients] can be switched to…although my experience of watching people being transferred to these drugs is it can take a while to get good blood pressure control…one of my concerns would be how safe it would be to switch people from drugs where we know there's a mortality benefit in heart failure and ischemic heart disease, to drugs where they don’t exist. So that would be my concern from a primary cardiac focus.”- C05

Abbreviations: ARR, aldosterone-renin ratio; GP, general practitioner; NSW, New South Wales; PA, primary aldosteronism.

Limited access to primary care in remote/regional areas was reported to result in delayed diagnosis and reactive specialist management. Overwhelmed specialist outpatient services with lengthy waiting lists further contributed to missed screening opportunities (Table 3, Q3.2). Many participants also discussed limited opportunity for follow-up, particularly in regional remote areas and in public hospital settings, which influenced screening and treatment decisions for clinicians (Table 3, Q3.3).

Resourcing in terms of time was a barrier for some participants to perform PA screening, relating to perception of burden/complexity of screening and confirmatory testing, as well as addressing PA in complex patients (Table S6, Q3.2). A small number of participants reported that equitable access to systems that could consolidate guidelines and streamline testing would improve the diagnostic process (Table S6, Q3.3).

#### Challenges Navigating Patient Preferences, Safety, and Cost

Participants reported several patient factors influencing access to PA diagnosis and treatment, broadly grouped into patient preferences and needs, safety or tolerability of procedures or treatments, and cost to patients.

Barriers relating to patient preferences and needs included concerns about radiation exposure (for computed tomography), medication side effects, opposition to surgery, reluctance to undergo procedures/treatment, and inconvenience of hospital admission for confirmatory testing (Table S6, Q3.4).

Concerns about adverse effects from spironolactone influenced treatment decisions for some clinicians, although most perceived it as well-tolerated and effective. Patient safety was a major concern reported by many clinicians, frequently limiting antihypertensive medication switching required for screening, resulting in reduced access to screening for comorbid and complex patients (Table 3, Q3.4).

Cost to patients for specialist care, primary care, medication switching, and spironolactone alternatives were also identified as barriers to timely PA diagnosis or optimal treatment (Table S6, Q3.5).

### Theme 4: Health Systems, Government, and Organizational Factors Underpin Screening and Treatment Decisions

Features of organizational and health service structures are instrumental in the ways in which PA screening, diagnosis, and treatment are delivered.

#### Health Systems, Personnel, and Patient Management

Integration of specialized services within hospitals provided access to gold standard PA diagnosis and treatment, available to some participants (Table S7, Q4.1). Specialized staff, including biochemists, support nurses, endocrine nurses, and radiologists to perform AVS, enabled timely and coordinated care in these units (Table 4, Q4.1). Outside of specialized units, disjointed care within the hospital context was reported, causing delays and lack of investigation for PA (Table S7, Q4.2). Limitations to ongoing follow-up and monitoring (Table S7, Q4.3), inconsistent referral practices (Table S7, Q4.4), and lack of longitudinal patient data capture and ongoing patient monitoring (Table S7, Q4.5) inhibited PA screening and appropriate management. Conversely, patient education and shared care models, especially collaboration with GPs for medication switching and follow-up, enabled successful PA management (Table 4, Q4.2).

**Table 4:** Theme 4 Representative Quotes

Theme 4: Health Systems, Government, and Organizational Factors Underpin Screening and Treatment Decisions		
Subtheme	Label	Quote
Health systems, personnel, and patient management	Q4.1	“We have a really good adrenal vein sampling set up at [metropolitan hospital]. We’ve got a single radiologist who’s very experienced at doing it, we have a lot of confidence in him. We also have…point of care cortisol testing when they’re having the adrenal vein sampling.”- E07
	Q4.2	“I do rely on their GPs to help when I need to pull [patients] off medication…What I don’t want is for them to then have inadequate control of their blood pressure through the process…I’ll liaise with the GP and say…can you do some blood pressure checks and adjust their medications accordingly?…[Several] weeks later, we’ll do the ARR and…saline suppression test.”- E07
Government funding for tests, services, and treatments	Q4.3	“All public hospitals in Queensland…they’ll pay for flights and accommodation [to access specialist services], so it makes accessing these services much, much easier…There are also…nurse navigators in this health system…[who] help try and chunk appointments together for people.”- G07
Communication and professional networks	Q4.4	“At the multidisciplinary team meeting, the AVS cases will be presented along with relevant imaging…It’s a team decision about whether it’s unilateral, bilateral or a nondiagnostic test, which is quite helpful for someone that doesn't arrange these tests often…Based on that, the multidisciplinary team meeting will come up with a suggestion, whether they need repeat AVS or surgery or medical management.”- E03
Professional role and culture	Q4.5	“For some patients I would overdo the [secondary hypertension] screening because they're coming to a renal clinic, they have seen the GP multiple times, the blood pressure is probably not under control when they come my way. So, I sometimes feel like I’m overdoing it, but more for the sake of completeness and maybe medicolegally not to miss a potential secondary cause…If I found a cause that’s an endocrine cause, and it’s something that I need some help with, I will activate endocrine…I think my role [in PA management] is more screening and referring.” - N10

Abbreviations: ARR, aldosterone-renin ratio; AVS, adrenal vein sampling; GP, general practitioner; PA, primary aldosteronism.

#### Government Funding for Tests, Services, and Treatments

Government-funded staff roles and systems enabled efficient and high-quality care to be accessed by patients: individual patient use systems enabled subsidies for medication not on the Pharmaceutical Benefits Scheme (eg, eplerenone) for patients unable to tolerate spironolactone; subsidized travel for remote patients, enabled access to services to facilitate PA diagnosis; and funding for nurse navigators, enabled appointment scheduling for rural remote patients (Table 4, Q4.3). However, funding limitation concerns were also expressed regarding Medicare subsidies for ARR; GPs reported fear of repercussion if including ARR more routinely in blood tests (Table S7, Q4.6). One participant expressed a view of the long-term economic benefit in early detection and treatment of PA (avoiding complication-driven treatment), whereas others viewed increasing PA screening as an added cost burden (Table S7, Q4.7).

#### Communication and Professional Networks

Many participants reported that effective inter-clinician communication supported screening and confirmatory testing decisions, BP monitoring for medication switching, and patient management for follow-up after discharge (Table S7, Q4.8). Professional networks developed via multidisciplinary teams and continuing professional development led to confidence and efficiency in decision making about patient management (Table 4, Q4.4). Personal, professional contacts were leveraged in referrals for further investigation or treatment (Table S7, Q4.9). However, isolation in clinical practice, often due to regional/remote location, was associated with reduced engagement with colleagues and a perception of being out of the loop with changes in practice and service provision outside of their location (Table S7, Q4.10).

#### Professional Role and Culture

Many participants expressed that their clinical roles, services available within their workplaces, and influence and input of colleagues shaped decision making in PA screening and management (Table S7, Q4.11). Many participants had clear boundaries around what was appropriate to investigate within their role versus the roles of other specialties (Table 4, Q4.5). Most participants viewed endocrinologists as the preferred specialty to manage PA investigation beyond ARR (Table S7, Q4.12) and perceived GPs as best positioned to perform community screening, while acknowledging barriers to achieve this.

## Discussion

This study provides the first cross-specialty qualitative analysis of factors influencing clinical decision making in PA screening and management. Clinician experience, knowledge, and perceptions, together with diagnostic complexity, resource availability, and government and organizational factors, all shaped decisions about screening for secondary hypertension, including PA, and influenced pathways to diagnosis and treatment.

Consistent with prior work showing that targeted education increases PA screening among GPs,^18^ here, recent or frequent exposure to PA strongly promoted screening and information seeking about PA. Conversely, limited exposure reduced clinicians’ proclivity to consider PA, even when aware of its prevalence, creating a vicious cycle in which underdiagnosis reinforces low detection. Interventions that can address limited awareness of, or reluctance to screen for PA, will be critical in addressing this barrier.

Many participants applied stringent criteria when selecting patients for PA screening, consistent with what has been described by others.^15^^,^^24^^,^^25^ Screening was typically limited to patients being young, taking multiple antihypertensive medications, or having evidence of electrolyte abnormalities or end-organ damage. Older or comorbid patients were often excluded due to concerns around safety, compliance, surgical eligibility, and the perceived marginal benefit of diagnosis. Some clinicians opted to treat empirically with MRAs rather than pursue diagnostic workup, adopting a pragmatic approach where resources were limited. However, adrenalectomy offers superior cardiovascular and kidney outcomes compared with MRA therapy.^26^, ^27^, ^28^, ^29^, ^30^ These data therefore highlight the need for additional clinician and health system supports to enable surgical management to be offered equitably to eligible patients.

Clinical guidelines were frequently referred to but may inadvertently discourage broader screening for PA. Earlier Australian and international guidelines recommend secondary hypertension screening only in specific high-risk populations,^31^^,^^32^ whereas recent European Society of Cardiology and Endocrine Society hypertension guidelines now suggest routine screening for PA in all individuals with hypertension.^33^^,^^34^ When stringent screening guidelines are adhered to, PA detection is markedly increased^35^ and is cost-effective,^36^ further supporting these updates. Translating these updated recommendations to Australian clinical practice remains a key implementation challenge.

Beyond deciding whom to screen, test complexity and clinical burden further influenced decision making. As reported by others,^18^^,^^24^^,^^37^ here, many participants found medication switching and ARR interpretation challenging, particularly in older and comorbid patients, contributing to screening avoidance. Many viewed the overall diagnostic pathway, including confirmatory testing and AVS, as burdensome. Compounded with limited access to primary care and diagnostic services, particularly outside metropolitan areas, this promoted triaging for investigation, diagnostic delay, and complication-driven investigation. Improving access to diagnostic services, as well as funding to embed specialized units with the capacity for efficient and reliable PA workup more broadly, will be critical to address these barriers.

This large qualitative study captures a wide range of clinician experiences, highlighting significant variability in PA management across Australia. However, this work represents only a snapshot of views and experiences within the Australian context. Although clinician perceptions of patient and health system factors were revealed, these data were not explicitly interrogated and may be addressed by future patient-centered and health system-focused studies.

In conclusion, given Australia’s limited health resources and geographic diversity, improvements in PA care must be practical and cost-effective. Our findings suggest several actionable opportunities (Box 2) and highlight the need to codesign interventions with clinicians to ensure relevance, feasibility, and sustainability. Addressing the identified barriers has the potential to substantially improve PA detection and patient outcomes.

Box 2 **Targeted Strategies to Improve PA Diagnosis and Management**

**Table:** 

Theme	Subtheme	Category	Target for Innovation
Theme 1: Clinician experience, knowledge, and perceptions guide clinical decision making in PA screening and clinical management	Educational sources and guidelines	Lack of information for clinicians	Advocacy and education about PA to promote awareness and increase screening
	Perceptions about PA, patients and other clinicians	Clinician perceptions about PA	
		Perception of patient health literacy and compliance	
	Clinical criteria and judgment for patient management	Patient characteristics and clinical information that inhibits secondary HTN or PA screening or PA workup	Updates to guidelines to include ARR screening more broadly
T2 – PA screening and diagnosis complexity and burden influences clinician decision making	Clinical guideline complexity, test reliability, and technical challenges	Clinical guideline complexity and challenges	
		Test reliability	Improvements in ARR assay reliability and requirements
	Patient complexity and medication switching	Medication switching burden	
		Medication switching complexity	
T3 – Accessibility of services and information influence clinical management	Access to medical facilities and services	Limited or lack of access to services	Improving access to screening and diagnostic workup for PA
		Limited access to primary care	
		Limited resourcing impacts screening and treatment decisions (triage) and outcomes	
T4 – Health systems, government, and organizational factors underpin screening, diagnosis, and treatment decisions	Health systems, personnel and patient management	Specialized services	Embedding gold standard workflow in hospitals
		Implementation facilitators	
	Communication and professional networks	Clinician–clinician and patient correspondence	Development of communities of practice and fostering interdisciplinary collaboration
		Organizational meetings

Abbreviations: ARR, aldosterone-renin ratio; HTN, hypertension; PA, primary aldosteronism.

## Supplementary Materials

Supplementary File (PDF) Item S1-S2; Table S1-S7
