# Supplementary material for: Clinician Decision Making in Managing Primary Aldosteronism: A Qualitative Study
Source: Kidney Med. 2026 Jun 12;8(9):101429. doi: 10.1016/j.xkme.2026.101429 (PMC13475217; doi:10.1016/j.xkme.2026.101429)
Supplement: Supplementary file 1 [file mmc1.docx]

# Item S1. Semi-structured interview guide

## General management of hypertension

1. Thoughts about diagnosing and managing hypertension:

- How has this changed over the years/ what influenced these changes?
- How confident do you feel diagnosing and managing it?

2. Can you talk me through how you screen/test/diagnose a typical patient with suspected hypertension?
   - What makes you initiate investigation of possible causes?

*Clinical criteria? Routine screening? BP uncontrolled with medication? Incidental findings?*

- Considerations when deciding what causes of hypertension to screen for?
  *Guidelines followed? Experiential observations? Established protocol?*

**If PA mentioned** – *what observations you usually associate with a candidate for PA screening?*

- How long before you typically begin screening?
- What makes you decide that a patient should not be investigated for a specific cause of hypertension?

*Lifestyle factors? Medication preference? Hypertension considered “normal”? Rarity?*

3. How much variation is there in your process? What causes this variation?

*Referral reason? Co-morbidities? Patient preferences?*

## Management of Primary Aldosteronism

4. Have you encountered a patient with suspected PA?

**If yes** – Can you talk me through how you managed that patient?

- What makes you initiate an investigation of PA?

*Clinical criteria? Routine screening? Uncontrolled BP with medication?*

*If non-GP, can you talk me through the patient pathway to you? E.g. info provided in referral, pre-testing*

- How long before you typically begin screening?
- What makes you decide that a patient should not be investigated for PA?

*Lifestyle factors? Medication preference? Hypertension considered “normal”? Rarity? Cost? disease severity? Why?*

5. Has your diagnostic process changed over time?

*What has prompted that, and why?*

6. **FOR PRIMARY CARE:** How does PA screening fit in to your normal workflow for HTN screening and management?
7. What do you see your role as, in PA screening and management?
8. Do you have personal connections to specialists/specialist clinics?

**If no, ask:**

- What do you know about PA?

- How would you screen/diagnose/treat a potential PA patient?

**then, SKIP to preamble before question 16**

## Screening and Diagnosis

9. Can you talk me through your testing process for PA? (Screening tests)
   - Why?
   - Pre-test actions?

*E.g. Change BP or hormonal medications or instructions to patients?*

*If medication switching is not done, why?*

- Opinion of tests used?

*E.g. complexity/usability, adaptability, cost, usefulness, accuracy, evidence base, barriers to use*

- Actions following receipt of results?

*E.g. interpretation (consideration of threshold), treatment choices, referrals for both positive and negative results*

*How are patients involved in the process of diagnosis? Preferences?*

10. For what tests would you refer patients?

- To whom?
- When? Why?
- Pre-test actions?	*E.g. Change BP or hormonal medications or instructions to patient?*
- Opinion of test?

*E.g. complexity/usability, adaptability, cost, usefulness, accuracy, evidence base, barriers to use?*

- Actions following receipt of results?

*E.g. interpretation (consideration of threshold), treatment choices, referrals for both positive and negative results*

11. Are there any other tests you are aware of but don’t use?
    - *Aldosterone to Renin Ratio*
    - *IV saline suppression testing*
    - *Fludrocortisone suppression testing*
    - *Captopril challenge testing*
    - *Adrenal CT*
    - *PA subtyping (AVS)*

**ASK:**

- Why don’t you refer for/perform/order this test?

## Treatment

12. Can you talk me through the main treatments you consider for PA and how you decide which is suitable for a particular patient?
    - When?

*E.g. frequency, stage of treatment*

- Why?

*e.g. experience, information source*

- Opinion of this treatment option?

*E.g. effectiveness, evidence base, complexity, adaptability, cost, barriers to use*

- *How do patients feel about the treatment options? Preferences, compliance*
- Opinion on MRAs compared to other HTN drugs?

*Why?*

13. For any of the following treatments not mentioned:
    - *Adrenalectomy*
    - *Mineralocorticoid receptor antagonists*

**ASK:**

- What do you know about the use of [insert treatment] in managing PA?
- Why don’t you use this treatment option?

14. What is the follow up process for a patient that is put on medical management?

## Potential improvements

15. How well do current screening/diagnosis/treatment methods meet patient needs?
    - *Confidence in diagnosing and treating PA?*

**SCRIPT - No prior experience or knowledge of PA:**

PA is a potentially curable cause of hypertension and is estimated to affect 3-14% of hypertensive patients in primary care, yet diagnosis rates are as low as 0.1% in Australia in primary care. The Primary Aldosteronism Centre of Excellence has been funded to address this gap and to increase the early diagnosis and treatment of PA to improve patient outcomes.

16. In an ideal world, how would PA be diagnosed and treated? *Why?*
17. What difference would this ideal scenario make?

- *Patient care?*
- *In your job role?*
- *Healthcare system?*
- *Others involved in patient management?*

18. What do you think is required to make this a reality?

- *For clinicians, clinics, patients, other supporting services?*

**Skip the two below if not currently possible (e.g. a new test) and consider asking “Are there any other changes that are possible now?”**

- Do you have agency and workplace support to do things differently to improve your work processes?
- Are you equipped with resources required for optimal PA diagnosis and treatment?

19. How does PA compare with other causes of hypertension you diagnose and treat on a regular basis?
    - *Ease and accuracy of diagnosis*
    - *Ease and effectiveness of treatment*
    - *Impact of diagnosis and treatment on patient’s quality of life*

## Barriers and Facilitators

20. How did you learn about PA?

**Or, if no prior PA knowledge:** how do you learn about HTN diagnosis/management?

- *Formal training, network of colleagues (who/what level), practice colleagues, clinical guidelines, opinion leaders*
- *Do you follow clinical guidelines when managing PA?*
- *How do you ensure you continually improve your practice? (e.g. formal feedback, autonomy, time)*

21. Your opinion about the existing guidelines for the diagnosis and treatment of PA?

- *Taken as a whole, your opinion of the PA diagnosis and treatment clinical pathway?*
- *Guideline developers, quality of the evidence for guidelines, adaptability to your setting, complexity of process, guideline design*

22. How do you think other [insert clinician type] diagnose and treat PA?
    - *Other colleagues at your practice?*
    - *Why are they different/the same?*
23. Anything else to add that would help us understand how to improve the diagnosis and treatment of PA?
24. Willing to be contacted for follow-up, if required (e.g. validation of results) and to receive results of the study?
25. Colleagues that may have more/less experience with PA management that may be interested in participating?

# Item S2. Full Methods

## Study design

A qualitative, exploratory study was chosen to allow in-depth investigation of clinicians’ experiences and perspectives on the barriers and enablers to screening, diagnosing and treating PA. A semi-structured interview guide was developed based on the updated Consolidated Framework for Implementation Research (CFIR) Interview Guide Tool^1^, a commonly used theoretical framework in implementation science comprising five domains associated with effective implementation: intervention characteristics, outer setting, inner setting, characteristics of individuals and implementation process^2,3^.

The study is reported in accordance with the Consolidated Criteria for Reporting Qualitative Research (COREQ)^4^ and was approved by the Monash University Human Ethics Committee (Project ID: 41728). Participants provided consent during completion of a screening survey and at the beginning of interviews. Participants received a $100 e-gift card as honorarium.

## Recruitment and sampling

Participants were recruited using purposive and snowball sampling. Inclusion criteria included endocrinologists, nephrologists, cardiologists and GPs practising in Australia. Exclusion criteria included involvement in PA research as a chief investigator, associate investigator, sponsor or participant within the past 5 years. Purposive sampling ensured diverse representation across key characteristics, including: (1) clinical specialty (2) practice location (remote/ regional/ metropolitan and state/ territory); (3) practice setting (public/ private/ mixed); and, (4) years of clinical experience. Sampling aimed for approximately 10 participants per clinical speciality group and continued until theoretical saturation was reached.

Participants were recruited via professional associations/societies, websites, social media channels and via practice-based research network (PBRN) mailing lists. Direct emails and phone calls to endocrinology, nephrology, cardiology and GP clinics were also made using publicly available information. Snowball sampling was achieved via interview participants and members of the Primary Aldosteronism Foundation and associated Facebook group. Study advertisements directed at people living with PA invited them to nominate a clinician for the study via a nomination survey.

Potential participants were invited to complete a screening survey collecting contact and demographic data to facilitate purposive sampling. Both the nomination and screening surveys were hosted on Qualtrics XM (Qualtrics, Provo, UT) and included a participant explanatory statement.

## Data collection and analysis

Semi-structured interviews were conducted by SH, a researcher with a background in biomedical and healthcare research, and LHJY, a researcher with qualitative research experience, via Zoom (Zoom © 2020) online video-conferencing from May to November 2024. Neither interviewer had established relationships with their interviewees. Before interviews, participants were informed of the interviewers’ role. Field notes were recorded after interviews, capturing key discussion points and interviewer reflections. No repeat interviews were conducted.

Interview transcripts were generated using Panopto (version 15.9.0.00022), checked for accuracy, de-identified and imported into NVivo (version 14) for analysis. Data analysis was conducted concurrently with data collection to enable refinement of the interview guide and purposive sampling. Two complementary qualitative analysis approaches were employed simultaneously. Deductive content analysis was performed by SH to code interview content against CFIR domains^1^ and Endocrine Society guidelines^5^. Reflective thematic analysis^6^ was undertaken by LHJY to inductively explore clinicians’ experiences and approaches to diagnosing and managing PA. Coding was documented and refined iteratively using Microsoft Excel and nVivo by SH and LHJY. Matrices were developed to systematically identify patterns within and between different participant groups. Regular meetings were held by SH and LHJY to compare and refine codes, facilitating integration across deductive and inductive approaches. To ensure trustworthiness, member checking was conducted by providing participants with a preliminary report for feedback. Audit trails were maintained through meeting minutes and post-interview field notes. Findings were reviewed by KL, an implementation science researcher with a qualitative research background, and the broader research team (GR, a primary care clinician and health services researcher, and JY, an endocrinologist and endocrine hypertension researcher with expertise in PA diagnosis and management) for expert review.

# Table S1. Participant Demographics

|  | **Cardiologists**  **n = 8** | **Endocrinologists**  **n = 10** | **GPs**  **n = 10** | **Nephrologists**  **n = 10** | **Total**  **n = 38** |
| --- | --- | --- | --- | --- | --- |
| **Clinical experience (years)** | | | | | |
| 1 – 5 | 1 (12.5) | 3 (30.0) | 2 (20.0) | 3 (30.0) | 9 (23.7) |
| 6 – 10 | 4 (50.0) | 5 (50.0) | 3 (30.0) | 5 (50.0) | 17 (44.7) |
| 11 – 15 | 2 (25.0) | 1 (10.0) | 1 (10.0) | 1 (10.0) | 5 (13.2) |
| 16 – 20 | - | 1 (10.0) | - | - | 1 (2.6) |
| 21 – 25 | - | - | 1 (10.0) | - | 1 (2.6) |
| 26 – 30 | - | - | 2 (20.0) | 1 (10.0) | 3 (7.9) |
| 31 – 35 | 1 (12.5) | - | 1 (10.0) | - | 2 (5.3) |
| **Practice type** | | | | | |
| Public only | 1 (12.5) | 3 (30.0) | 1 (10.0) | 6 (60.0) | 11 (28.9) |
| Public and private | 6 (75.0) | 5 (50.0) | 3 (30.0) | 3 (30.0) | 17 (44.7) |
| Private only | 1 (12.5) | 2 (20.0) | 6 (60.0) | 1 (10.0) | 10 (26.3) |
| **Location (state)** | | | | | |
| ACT | - | - | - | 1 (10.0) | 1 (2.6) |
| NSW | 1 (12.5) | 4 (40.0) | 3 (30.0) | 4 (40.0) | 12 (31.6) |
| NT | - | 1 (10.0) | - | - | 1 (2.6) |
| QLD | - | - | 3 (30.0) | 1 (10.0) | 4 (10.5) |
| SA | - | 2 (20.0) | 1 (10.0) | 1 (10.0) | 4 (10.5) |
| TAS | 3 (37.5) | - | - | - | 3 (7.9) |
| VIC | 4 (50.0) | 2 (20.0) | 2 (20.0) | 3 (30.0) | 11 (28.9) |
| WA | - | 1 (10.0) | 1 (10.0) | - | 2 (5.3) |
| **ASGS classification*** | | | | | |
| Major cities RA1 | 4 (50.0) | 6 (60.0) | 7 (70.0) | 8 (80.0) | 25 (65.8) |
| Inner regional RA1 | - | - | 1 (10.0) | - | 1 (2.6) |
| Inner regional RA2 | 4 (50.0) | 3 (30.0) | - | 2 (20.0) | 9 (23.7) |
| Outer regional RA3 | - | - | 2 (20.0) | - | 2 (5.3) |
| Remote Australia RA4 | - | 1 (10.0) | - | - | 1 (2.6) |

Note: Demographic profile of participants. Numbers in parentheses represent percentages. ACT, Australian Capital Territory; NSW, New South Wales; NT, Northern Territory; QLD, Queensland; SA, South Australia; TAS, Tasmania, VIC, Victoria; WA, Western Australia; ASGS, Australian Statistical Geography Standard.*^7^

# Table S2. Coding density to the CFIR framework

|  | Cardiologists | Endocrinologists | GPs | Nephrologists |
| --- | --- | --- | --- | --- |
| I. A. Innovation Source | 1 | 1 | 2 | 0 |
| I. B. Innovation Evidence-Base | 7 | 8 | 6 | 8 |
| I. C. Innovation Relative Advantage | 7 | 8 | 3 | 10 |
| I. D. Innovation Adaptability | 0 | 0 | 0 | 0 |
| I. E. Innovation Trialability | 0 | 0 | 0 | 0 |
| I. F. Innovation Complexity | 8 | 10 | 5 | 10 |
| I. G. Innovation Design | 2 | 9 | 4 | 1 |
| I. H. Innovation Cost | 3 | 2 | 4 | 2 |
| II. A. Critical Incidents | 0 | 0 | 0 | 0 |
| II. B. Local Attitudes | 0 | 0 | 0 | 0 |
| II. C. Local Conditions | 0 | 0 | 0 | 0 |
| II. D. Partnerships & Connections | 3 | 5 | 0 | 2 |
| II. E. Policies & Laws | 0 | 0 | 3 | 0 |
| II. F. Financing | 2 | 6 | 1 | 2 |
| II. G. External Pressure | 0 | 0 | 0 | 0 |
| III. A. Structural Characteristics | 5 | 3 | 1 | 3 |
| III. B. Relational Connections | 7 | 9 | 8 | 6 |
| III. C. Communications | 2 | 5 | 2 | 2 |
| III. D. Culture | 7 | 8 | 9 | 10 |
| III. E. Tension for Change | 0 | 1 | 2 | 4 |
| III. F. Compatibility | 3 | 4 | 3 | 6 |
| III. G. Relative Priority | 6 | 3 | 3 | 7 |
| III. H. Incentive Systems | 1 | 1 | 2 | 1 |
| III. I. Mission Alignment | 2 | 3 | 1 | 3 |
| III. J. Available Resources | 6 | 9 | 8 | 7 |
| III. K. Access to Knowledge & Information | 8 | 10 | 10 | 10 |
| IV. 2. A. High-level Leaders | 2 | 6 | 1 | 3 |
| IV. 2. B. Mid-level Leaders | 0 | 0 | 0 | 0 |
| IV. 2. C. Opinion Leaders | 0 | 0 | 0 | 0 |
| IV. 2. D. Implementation Facilitators | 0 | 5 | 1 | 2 |
| IV. 2. E. Implementation Leads | 0 | 0 | 0 | 0 |
| IV. 2. F. Implementation Team Members | 0 | 0 | 0 | 0 |
| IV. 2. G. Other Implementation Support | 0 | 0 | 0 | 0 |
| IV. 2. H. Innovation Deliverers | 8 | 10 | 10 | 6 |
| IV. 2. I. Innovation Recipients | 8 | 10 | 8 | 4 |
| IV. 1. A. Need | 8 | 10 | 9 | 10 |
| IV. 1. B. Capability | 7 | 9 | 10 | 8 |
| IV. 1. C. Opportunity | 8 | 10 | 10 | 9 |
| IV. 1. D. Motivation | 5 | 9 | 8 | 8 |

Note: Coding density to CFIR sub-domains within domains I – IV, shown for each clinical speciality. Values represent the number of participants that expressed ideas that were coded to sub-domains.

# Table S3. Coding density to the inductive thematic framework.

|  | Cardiologists | Endocrinologists | GPs | Nephrologists |
| --- | --- | --- | --- | --- |
| T1 - 1.1.1. Clinical experience informs practice and further education | 1 | 5 | 6 | 7 |
| T1 - 1.1.2. Limited clinical exposure to PA or full workup to adrenalectomy | 7 | 4 | 10 | 7 |
| T1 - 1.1.3. Positive clinical experience with procedure or treatment | 4 | 6 | 1 | 7 |
| T1 - 1.2.1. Reference to clinical guidelines or online resources | 7 | 9 | 10 | 10 |
| T1 - 1.2.2. Education source | 8 | 10 | 8 | 8 |
| T1 - 1.2.3. Influential figures | 2 | 6 | 1 | 3 |
| T1 - 1.2.4. Lack of information for clinicians | 2 | 2 | 4 | 4 |
| T1 - 1.3.1. Clinician perceptions about PA | 3 | 3 | 9 | 4 |
| T1 - 1.3.2. Perception of patient health literacy and compliance | 4 | 6 | 3 | 4 |
| T1 - 1.3.3. Perception of other clinicians | 7 | 7 | 4 | 7 |
| T1 - 1.4.1. Patient characteristics and clinical information that inhibits secondary HTN or PA screening or PA workup | 6 | 6 | 6 | 8 |
| T1 - 1.4.2. Patient characteristics and clinical information that promote secondary HTN or PA screening or PA workup | 8 | 9 | 8 | 10 |
| T1 - 1.4.3. Routine practice for clinicians in secondary HTN screening | 5 | 5 | 6 | 6 |
| T2 - 2.1.1. Clinical guideline complexity and challenges | 3 | 9 | 4 | 2 |
| T2 - 2.1.2. Test reliability | 5 | 10 | 3 | 8 |
| T2 - 2.1.3. Technical challenges | 2 | 6 | 1 | 5 |
| T2 - 2.1.4. Test or procedure is acceptable or uncomplicated | 4 | 8 | 2 | 7 |
| T2 - 2.2.1. Patient co-morbidity and polypharmacy | 6 | 4 | 3 | 6 |
| T2 - 2.2.2. Variability in patient clinical manifestation and circumstances | 0 | 2 | 1 | 1 |
| T2 - 2.2.3. Medication switching burden | 1 | 3 | 2 | 4 |
| T2 - 2.2.4. Medication switching complexity | 6 | 5 | 2 | 6 |
| T2 - 2.3.1. Advantage of PA diagnosis (general) | 3 | 4 | 3 | 5 |
| T2 - 2.3.2. Advantage of PA diagnosis for younger patients | 1 | 0 | 0 | 2 |
| T2 - 2.3.3. Conditions must be met for PA screening or workup to proceed | 3 | 6 | 0 | 7 |
| T2 - 2.3.4. Perception of limited benefit in PA screening or workup | 6 | 6 | 2 | 7 |
| T2 - 2.3.5. Adverse events promote PA investigation and treatment | 0 | 0 | 1 | 1 |
| T3 - 3.1.1. Ease of access to services | 2 | 4 | 5 | 4 |
| T3 - 3.1.2. Limited or lack of access to services | 5 | 7 | 5 | 4 |
| T3 - 3.1.3. Limited access to primary care | 1 | 4 | 1 | 1 |
| T3 - 3.1.4. Limited resourcing impacts screening and treatment decisions (triage) and outcomes | 0 | 3 | 3 | 3 |
| T3 - 3.2.1. Referrals and follow up | 5 | 6 | 5 | 4 |
| T3 - 3.1.5. Time allocation | 2 | 1 | 2 | 2 |
| T3 - 3.1.6. Accessibility of guidelines and resources | 1 | 0 | 2 | 0 |
| T3 - 3.2.2. Patient preferences and needs | 4 | 6 | 4 | 2 |
| T3 - 3.2.3. Safety and tolerability of procedures or treatments | 7 | 7 | 2 | 6 |
| T3 - 3.2.4. Cost to patient | 3 | 3 | 3 | 1 |
| T4 - 4.1.1. Specialised services | 3 | 5 | 0 | 2 |
| T4 - 4.1.2. Implementation facilitators | 0 | 5 | 1 | 2 |
| T4 - 4.1.3. Disjointed care and delayed diagnosis | 3 | 3 | 1 | 3 |
| T4 - 4.1.4. Patient follow up in hospital setting | 3 | 2 | 0 | 2 |
| T4 - 4.1.5. Challenges in patient management | 3 | 2 | 3 | 2 |
| T4 - 4.1.6. Procedural facilitators in PA management | 4 | 8 | 7 | 4 |
| T4 - 4.2.1. Services that facilitate PA diagnosis and management | 0 | 3 | 1 | 1 |
| T4 - 4.2.2. Medicare and PBS related barriers to PA diagnosis and management | 2 | 6 | 3 | 1 |
| T4 - 4.2.3. Economic factors | 1 | 1 | 1 | 1 |
| T4 - 4.3.1. Clinician - clinician and patient correspondence | 5 | 5 | 3 | 4 |
| T4 - 4.3.2. Organisational meetings | 2 | 6 | 1 | 3 |
| T4 - 4.3.3. Referrals | 2 | 1 | 2 | 2 |
| T4 - 4.3.4. Limited communication between clinicians | 1 | 1 | 0 | 0 |
| T4 - 4.3.5. Isolation in clinical practice | 3 | 1 | 3 | 3 |
| T4 - 4.4.1. Workplace exposure and clinical role | 3 | 4 | 3 | 6 |
| T4 - 4.4.2. Perceptions of the role of other specialties in PA management | 7 | 7 | 3 | 7 |
| T4 - 4.4.3. Assumed roles in PA diagnosis and management | 7 | 4 | 9 | 8 |

Note: Coding density to Inductive sub-domains within themes 1 - 4, shown for each clinical speciality. Values represent the number of participants that expressed ideas that were coded to sub-themes.

# Table S4. Theme 1 representative quotes

| Subtheme | Label | Quote |
| --- | --- | --- |
| Clinical Exposure to PA | Q1.1 | "I have to say, I haven't [kept up to date with PA knowledge], only because I haven't found, recently, a patient that has had it. But I think if I do find someone that has it, that would be the trigger for my reading around it. I mean, I've encountered it before, it's just I haven't encountered it myself recently." - E04 |
|  | Q1.2 | "I've been working as a cardiologist for probably 14 years now, and I've picked up one secreting adenoma which is like, that's the holy grail. I guess that's why you're testing for it…That person has had their adenoma removed, and their blood pressure is fixed which is really good...but that's out of a lot of tests to find one that's led to a really useful intervention." - C03 |
| Educational sources and guidelines | Q1.3 | "If I get to the pointy end...the referral pathway for adrenal vein sampling is still not clear. It seems to change a bit, and it changes in [the closest major city], and I'm a long way from [there], so we don't get told any of the changes. So that, I think, is the biggest challenge really, is that the final step of testing is a little bit outside of our control and our knowledge..." - N03 |
| Perceptions about PA, patients and other clinicians | Q1.4 | "During my physician training there was the SPRINT trial that suggested, in general, people are not being adequately treated for their blood pressure. And since then, there's been another couple of papers that suggest that we in particular were underdiagnosing hyperaldosteronism as a cause. The European society tends to be what I follow in general as a cardiologist, and the 24 hours guidelines suggested that with resistant hypertension, most of the time it's actually an element of hyperaldosteronism. So, I'm screening for that a little bit more than I used to. And the other thing I'm doing more than I used to is actually bringing people off their medications to screen appropriately." - C06 |
|  | Q1.5 | "It's one of those things, like most of endocrinology, where the consequences of these decisions [to pursue diagnosis] are mostly intangible for patients. And so, telling them that they've got a slightly increased cardiovascular risk, doesn't change their emotional response to it, or their motivation to do something similar. It's like, well, I feel fine, my blood pressure's good, move on. Which is every single condition I manage, really." - E06 |
|  | Q1.6 | "I think one of the problems as well is that in primary care I've noticed a change in the 15 years that I've been in Australia, which is the amount of bulk billing practices has increased dramatically. So that means that the amount of time per patient has fallen. And therefore, I see that GPs [have] become very algorithm driven, because they don't have a lot of time and they're very single problem orientated.”- C05 |
|  | Q1.7 | "I think younger GPs like me would screen for [PA] more, because they're scared as hell of missing something. I think old school GPs that are lulled in clinical practice might be forgetting about it..." - G07 |
| Clinical criteria and judgement for patient management | Q1.8 | "If they are very hypertensive, over 160 consistently, or if they're quite young, with no other clear reason to be hypertensive, then I will often send off at least a half-hearted secondary hypertension screen. So, my threshold is maybe under 50 [years of age] with significant hypertension and no other reasons that they might be hypertensive. So if they're not significantly obese or have issues with obstructive sleep apnoea or have demonstrated unhealthy behaviours for a long period of time, if they're otherwise healthy and they have significant hypertension, I'll often do a screen... if they haven't started medication yet, I'll send off blood, like morning cortisol, I’ll send off a renin [aldosterone] ratio, I'll do renal tract Doppler ultrasounds, and then I'll often do a quick screen for secondary organ effects of hypertension..." - C01 |

# Table S5. Theme 2 representative quotes

| Subtheme | Label | Quote |
| --- | --- | --- |
| Clinical guideline complexity, test reliability and technical challenges | Q2.1 | "In general, I find [the guidelines to be] a bit long. Most guidelines are written by a committee, so I do think that in general, guidelines should be as comprehensive as they need to be. It would be good to have a flow chart or diagram, that sort of thing. The ESC always do that for complex cardiac issues, they'll have a key illustration. I think the European Society of Hypertension Practice guidelines don't have the same thing. There's a few diagrams and things in there, but they're really not as useful as they think they are in terms of investigations. It's mostly on when to consider screening and when to start treatment. When and who to consider for secondary hypertension screening, I'd say, is not as clearly defined for people." - C06 |
|  | Q2.2 | “I think the endocrine guidelines are good. I think the GP guidelines are pretty good, actually. I just don't think people follow them. I think for the endocrine ones...I think they're easy, they're well written, they make sense. They're just very cookie cutter in approach which is what a guideline is, right? It's what you do for most people. So, taking that with our knowledge of physiology and knowledge of the patient and their situation needs to be applied as well. But I think generally speaking they're all pretty good." - E09 |
|  | Q2.3 | "It's nice when you get a clear answer [for AVS], but again, there's often a lot of grey and it's incredibly frustrating when there's difficulty with cannulation or they have to go back for testing because of there's such a long lag time to get these investigations in my location." - E03 |
|  | Q2.4 | “And the truth in reality, is when we do the secondary hypertension work up, we just send it as a bulk, in the sense that we wouldn't just test for primary aldosteronism, and not do for hypercortisolism or pheochromocytoma, because often the tests are usually done in a very similar urine collection and blood test...So, you screen all in one go, with the understanding that you're obviously aiming for one diagnosis more than the other, with primary aldosteronism." - N07 |
| Patient complexity and medication switching | Q2.5 | "It's really just the preparation. It's switching the antihypertensives, often in people who are already high risk, they may have had strokes, that's the sort of population I'm seeing, they've got type two diabetes, they might have multiple medical conditions, they're on lots of medications. You've got to think about interactions with other medications, of the new antihypertensive you're using, of the risk of postural hypertension with Prazosin, and their capacity to understand how to self-adjust, which depending on their educational level, literacy, may be unreliable for them to self-titrate. And then just access to services, it's hard to get into GPs around here... it really relies on the patient or already stretched services to monitor their blood pressure during that time they're off their meds." - E02 |
| Burden versus advantage in pursuing PA diagnosis | Q2.6 | "I think it depends on the patient. You know the risk profile for surgery and mid to long term benefits. It's very different if you have primary aldosteronism and you are very young and don't have many medical problems and you don't want to be on medication forever...if it's a single solution where you can just do surgery and get rid of it...then I think it's very reasonable to do it…But if we’re talking of people who...surgery is going to be risky for them, and they have a shorter lifespan...going forward, because of other things, then you question, do you want to treat them with surgery? It depends a lot on the local skills of being to do the surgery and the testing, but then, is there much of a benefit? Because if they have heart failure, they're going to be on spironolactone anyway. Then you go, well, is there a benefit of doing surgery if you will put them on spironolactone anyway? It sort of it tips the balance." - N07 |
|  | Q2.7 | "The [patients] that require [adrenalectomy] are usually so dramatically hypertensive, they've had numerous…presentations to E.D. Some of them might have even had a stroke from it." - N04 |

# Table S6. Theme 3 representative quotes

| Subtheme | Label | Quote |
| --- | --- | --- |
| Access to medical facilities and services | Q3.1 | "I did everything that I could do for this patient, the CT, but my hospital doesn't have a good service to do the adrenal vein sampling and the [saline] suppression test. And so, I need someone else to help me with that. But I think now, I need to work out how to do it myself. Because the other hospital that I referred to, a quaternary referral centre...just hasn't done what I asked them to do." - C02 |
|  | Q3.2 | "If we had a blood test that I could... just send it off without having to fiddle with the medications and all of that, [it] would be ideal. Because I think that's the main barrier. I think, looking at my colleagues, I reckon a lot of them don't do [ARR] because it is a bit of a pain in the butt, it takes time and we are all very time pressured. And it even just takes time to explain to a patient what you're going to do and why, why do you care about my blood pressure? This can take a couple of minutes. And then, I need you to stop your medications, it's another couple of minutes... it adds up when you've only got 15 minutes to see someone from start to finish, that includes your history taking, your examination and interpretation of the investigations that they've already provided you with." - C06 |
|  | Q3.3 | "Normally we have a lot of external guidelines from various agencies of different diseases, so it's very fragmented... Once we have perhaps a level of AI that actually interacts with the software and actually analyses...and incorporates the measurements we make within the examination findings and then make specific suggestions to help us...I think that's going to improve the screening rate for things like PA." - G11 |
| Challenges navigating patient preferences, safety and cost | Q3.4 | "It's just about, why should I do this? My doctor has already investigated, my blood pressure is reasonably well controlled on this, why do I have to actually do this? So, to try to convince that this is the problem...I find it hard to justify them stopping this medication for maybe 2 or 4 weeks and then buying new medications. And most of them are apprehensive about what kind of side effects [they can] expect from this new medication. They are really apprehensive of that. So, I find it tricky to ask them to actually change it and switch over for a test. And most of them are on NSAIDs for muscle aches and pains and then I have to ask them, don't take that for four weeks. And I think sometimes it's a bit trickier in that cohort, unless they're very motivated about their health." - G05 |
|  | Q3.5 | "So I don't do a lot of private, but...one of my colleagues...who runs nice flashy private rooms, they charge a gap, it's not huge. The gap for a new consult is about $140. So, it's significant, but it's not enormous compared to some other areas, and they’re dead quiet, like to the point that they thought that they were going to go broke…I think it just reflects the fact that people are now not in a position to pay these gaps. They all come and see us, where really they could probably go and see a cardiologist once or twice in private, be all done in a couple of weeks and be sorted." - C03 |

# Table S7. Theme 4 representative quotes

| Subtheme | Label | Quote |
| --- | --- | --- |
| Health systems, personnel, and patient management | Q4.1 | "The more work that [Influential figure 1] has done, raising awareness and having the endocrine hypertension clinic, has meant that I look at it more often, because there's more of a pathway now for referring people for further investigation. Whereas earlier on, if I identified someone I was concerned about, it's just a lot of work. We didn't do saline suppression tests here, and there wasn't a very clear referral pathway before the endocrine hypertension clinic." - E02 |
|  | Q4.2 | "I work also with the Department of health on referral access criteria. We're trying to streamline referrals to hospitals, and whilst in a perfect world we'd be able to pick up the relevant information in a timely way, that's not always how the clinical course presents itself, and it certainly isn't how the system structure is organised. So a more comprehensive system might be that there is a two week turnaround in patients being seen and organised to a clear workflow, for how to diagnose and investigate primary hyperaldosteronism, and to decide how to move patients forward in the treatment of that. But of course, we need services to support that." - G10 |
|  | Q4.3 | "Now evidence [suggests that PA] can evolve...the tricky thing is we don't technically, in the public hospital systems, have a huge capacity to just keep seeing people ongoing. So, in my cases, I put them on the drug, I make sure that the renin is unsuppressed, the potassium is okay, they're not getting side effects, that they’re stable on the drug, that they're happy with it, that I'm happy with it. And then it's...individualised in how they do this, whether or not I see them on an annual basis for a bit, and check that things are still okay, or whether or not I refer them back to their GP...The problem in public is, we really do not have the ability to keep these people in our clinics year on year." - E07 |
|  | Q4.4 | "The hypertension clinic was really good because it was staffed by a nephrologist, a couple of cardiologists, a GP with an interest in hypertension and an endocrinologist. So it was perfect, and we could discuss cases and move cases between us if there was a particular need to. Someone comes in with really high blood pressure and angina they would come to me, if someone came in with renal dysfunction and hypertension they would go to one of the nephrologists. So that was really, probably an ideal model. How does it work otherwise? It's super random. It will entirely depend on where the GP refers to, if they put renal on there, they'll go to the renal clinic. If they put cardiology on, they'll come to us. And again, the reality is most of them now will sit on our category 3 wait list indefinitely." - C03 |
|  | Q4.5 | "I was concerned, particularly over the COVID pandemic, about how many consultations we were doing by telehealth and phone and not actually getting to measure blood pressure. And whilst we obviously moved largely back to in-person consults, there's still areas where we see patients for months at a time over telehealth. I know that particularly in rural and regional areas, sometimes there's a lot being done via telehealth. And I do worry with hypertension, which relies on a physical exam so much, that sometimes we may be misled in practice and not doing enough serial monitoring of blood pressure, so that's and that's something to think about." - E01 |
| Government funding for tests, services and treatments | Q4.6 | "[Medicare doesn't] like GP's to do medication that they expect specialists to do. The thing is, if I suspect somebody had coronary artery disease, I can send them for a stress test and then I would stop there after that...if I do the renin aldosterone test occasionally, then that probably won't cause a problem. But if I want to do more, [Medicare] will question me...especially because secondary hypertension is a condition that the government or health department [considers] shared care."- G06 |
|  | Q4.7 | "So definitely we may include [ARR] in the routine check up, however, the European Society of cardiology guideline of hypertension does not recommend [it as] first line...So definitely we do follow that practice...if we do this test for every patient, on the initial visit it might not be covered by Medicare, so that will be extra burden on the patient. And this needs to be paid out of pocket... there are plenty of tests you have to do every day for patients, so there will be extra burden on the system. This is what I believe." - G01 |
| Communication and professional networks | Q4.8 | "Well, if [the ARR] was extremely high, I would probably give someone a call, so either nephrology or endocrinology just to sort of ask for some guidance as to what to do. Because all I would say is if it's normal or abnormal. And if it's extremely abnormal and I know that the test has been done correctly, so off all the required medications, then I would be seeking another specialty’s, either renal or endo, input as to what to do." - C01 |
|  | Q4.9 | "Well, there's half a dozen endocrinologists in [this suburb]. And there's a couple of endocrine surgeons…and now there's one or two in [this suburb]. So, we all know each other, we work closely together, we cross refer. You know, even a phone call. We are all colleagues, no one's worried about ego or taking business from each other. We are a very good group in [this suburb]." - N04 |
|  | Q4.10 | "I'm in a bit of a silo here in the public hospital because I'm the only endocrinologist here. [Primary aldosteronism] hasn't come up in conversation much with the other endocrinologists in the region. That's not to say they're not investigating and diagnosing and treating...you do feel separated when you're out in the country...In [a major city], I was attending face to face meetings all the time, and you just get these informal conversations that would organically develop and relationships that were developed, and so you felt part of a larger community..." - E06 |
| Professional role and culture | Q4.11 | "I usually handover to endocrine for management. And here at [metropolitan hospital] I send to them earlier, [since] they have a very good endocrine service here. They do the confirmation test, the saline test. And [Influential figure 3]...has a special interest in PA so that's why here, if I encounter a case that has a high degree of suspicion of PA, I refer to endocrine. Where I worked previously, at [metropolitan hospital], I used to request the saline suppression test myself...I used to have a protocol that I would follow, and that's probably a bit biased because one of our colleagues has special interest in PA and did his PhD with [Influential figure 4]...So I can also order and interpret the saline suppression tests. But here, because of the ease of referring to endocrine I just send them in." - N10 |
|  | Q4.12 | "When we try to diagnose and investigate primary aldosteronism, we're not sure quite what we're dealing with and we're talking about specific assays and different investigations that are much more frequently addressed by the endocrinologists than by cardiologists. And I feel that [the endocrinologists] level of familiarity and engagement with the laboratory as well where the tests are being done, is greater. And I feel it's sensible that the specific issue is dealt with by the craft groups that have the greatest expertise in that area." - C07 |

# References

1.	Damschroder LJ, Reardon CM, Widerquist MAO, Lowery J. The updated Consolidated Framework for Implementation Research based on user feedback. *Implementation Science.* 2022;17(1):75.

2.	Kirk MA, Kelley C, Yankey N, Birken SA, Abadie B, Damschroder L. A systematic review of the use of the Consolidated Framework for Implementation Research. *Implementation Science.* 2016;11(1):72.

3.	Skolarus TA, Lehmann T, Tabak RG, Harris J, Lecy J, Sales AE. Assessing citation networks for dissemination and implementation research frameworks. *Implementation Science.* 2017;12(1):97.

4.	Tong A, Sainsbury P, Craig J. Consolidated criteria for reporting qualitative research (COREQ): a 32-item checklist for interviews and focus groups. *International Journal for Quality in Health Care.* 2007;19(6):349-357.

5.	Funder JW, Carey RM, Mantero F, et al. The Management of Primary Aldosteronism: Case Detection, Diagnosis, and Treatment: An Endocrine Society Clinical Practice Guideline. *J Clin Endocrinol Metab.* 2016;101(5):1889-1916.

6.	Braun V, Clarke V. Reflecting on reflexive thematic analysis. *Qualitative Research in Sport, Exercise and Health.* 2019;11(4):589-597.

7.	Australian Bureau of Statistics. Australian Statistical Geography Standard (ASGS) Edition 3. <https://www.abs.gov.au/statistics/standards/australian-statistical-geography-standard-asgs-edition-3/jul2021-jun2026>. Published 2021 - 2026. Accessed 7 May 2025.
